# Recent advances in understanding congenital myopathies

**DOI:** 10.12688/f1000research.16422.1

**Published:** 2018-12-11

**Authors:** Gianina Ravenscroft, Robert J. Bryson-Richardson, Kristen J. Nowak, Nigel G. Laing

**Affiliations:** 1 Centre for Medical Research, The University of Western Australia, Perth, WA, Australia; 2 Harry Perkins Institute of Medical Research, QEII Medical Centre, Nedlands, WA, Australia; 3 School of Biological Sciences, Monash University, Melbourne, VIC, Australia; 4 School of Biological Sciences, Faculty of Health and Medical Sciences, The University of Western Australia, QEII Medical Centre, Nedlands, WA, Australia; 5 Office of Population Health Genomics, Western Australian Department of Health, East Perth, WA, Australia; 6 Department of Diagnostic Genomics, PathWest Laboratory Medicine, QEII Medical Centre, Nedlands, WA, Australia

**Keywords:** congenital myopathy; carrier screening; genetics; skeletal muscle; therapies; molecular diagnosis; genetic technology

## Abstract

By definition, congenital myopathy typically presents with skeletal muscle weakness and hypotonia at birth. Traditionally, congenital myopathy subtypes have been predominantly distinguished on the basis of the pathological hallmarks present on skeletal muscle biopsies. Many genes cause congenital myopathies when mutated, and a burst of new causative genes have been identified because of advances in gene sequencing technology. Recent discoveries include extending the disease phenotypes associated with previously identified genes and determining that genes formerly known to cause only dominant disease can also cause recessive disease. The more recently identified congenital myopathy genes account for only a small proportion of patients. Thus, the congenital myopathy genes remaining to be discovered are predicted to be extremely rare causes of disease, which greatly hampers their identification. Significant progress in the provision of molecular diagnoses brings important information and value to patients and their families, such as possible disease prognosis, better disease management, and informed reproductive choice, including carrier screening of parents. Additionally, from accurate genetic knowledge, rational treatment options can be hypothesised and subsequently evaluated *in vitro* and in animal models. A wide range of potential congenital myopathy therapies have been investigated on the basis of improved understanding of disease pathomechanisms, and some therapies are in clinical trials. Although large hurdles remain, promise exists for translating treatment benefits from preclinical models to patients with congenital myopathy, including harnessing proven successes for other genetic diseases.

## Introduction

Congenital myopathies are typically characterised by hypotonia, skeletal muscle weakness, and specific pathological hallmarks on skeletal muscle biopsy (reviewed in 1). They are broadly grouped on the basis of the predominant pathological feature, specifically the presence of cores (core myopathy), central nuclei (centronuclear myopathy), or nemaline bodies (nemaline myopathy). Traditionally, diagnostic work-up and research of cases occurred following extensive clinical evaluation and muscle biopsy. This is changing, as genetic testing is increasingly the primary diagnostic approach.

Genetic diagnosis has improved with many novel disease-causing genes and variants causing congenital myopathies identified following the widespread adoption of massively parallel sequencing, and there was a peak in gene discovery in the early 2010s ^2^. Whilst the rate of gene discovery is slowing, one growth area in congenital myopathy genetics has been the identification of recessive congenital myopathies associated with pathogenic variants in genes previously associated only with dominant disease, namely *CACNA1S*, *SCN4A*, *TNNT3*, and *TTN.* Cases of congenital myopathy presenting *in utero* (sometimes as early as the first trimester with foetal akinesia and associated abnormalities, including multiple joint contractures, and arthrogryposis) are also increasingly recognised ^3^.

Alongside the advances in understanding the genetic basis, there has been a recent focus on genetic therapies for congenital myopathies, including exon skipping, RNA interference, and adeno-associated virus (AAV)-mediated gene replacement. The drive for therapies is complemented by increasing interest in the introduction of population-based carrier screening for recessive and X-linked diseases, including relevant congenital myopathies.

Here, we discuss the recent advances made toward understanding the molecular basis of and potential therapies for congenital myopathies, together with the current and future challenges for the field.

## Advances in understanding the genetic basis of congenital myopathies

The extensive gene discovery success over the last eight years allows the provision of a molecular diagnosis to many more patients with congenital myopathy than was previously possible ^4^. It is difficult to be certain of the overall diagnostic rate for congenital myopathies, but Agrawal *et al*. suggested that 60% to 80% of centronuclear myopathies were genetically resolved ^5^, and in a recent Danish study of 107 national congenital myopathy cases that were older than five years of age, 56% received a genetic diagnosis ^6^. Interestingly, the rate of genetic diagnosis was 83% in cases with specific features on skeletal muscle biopsy but only 29% in cases with non-specific histology ^6^. *RYR1* mutations are the most frequent culprit in congenital myopathy ^7^. However, despite targeted gene panel and whole exome sequencing, many patients with congenital myopathy remain without a genetic diagnosis.

In the last couple of years, six new congenital myopathy disease genes have been identified ( Table 1). In addition to contributing to improved diagnostics, the discovery of mutations in *PPA2* and *PYROXD1* adds altered redox regulation as a primary disease mechanism in the congenital myopathies. Despite these recent discoveries, the identification of novel disease genes has waned, suggesting that genetically undiagnosed families may harbour variants in known congenital myopathy disease genes that are not currently recognised as pathogenic or are in genes that represent very rare forms of disease. This renders the ability to identify and confirm further novel disease genes more difficult, since researchers typically rely on the identification of additional families with a similar phenotype and variants in the same gene to confirm the diagnosis in the initial family.

**Table 1.** Novel congenital myopathy disease genes, 2015–2018.

Gene	Findings	References
*MYL1*	Recessive loss-of-function variants were identified in two probands with severe myopathy characterised by loss of hypotrophic type II myofibres on biopsy.	8
*MYO18B*	Recessive variants were identified in a patient with nemaline myopathy and cardiomyopathy and in a family presenting with Klippel–Feil anomaly and myopathy.	9, 10
*MYPN*	Recessive loss-of-function mutations were associated with childhood onset, slowly progressive myopathy with nemaline bodies (including intranuclear rods), and caps on skeletal muscle biopsy. Patient biopsies showed a substantial reduction in myopalladin. Some patients also presented with cardiac involvement.	11, 12
*PPA2* ^a^	Recessive variants are associated with sudden cardiac death in infants and young adults. Skeletal muscle from one mildly myopathic infant displayed nemaline bodies.	13, 14
*PYROXD1*	Recessive variants were identified in five families in which affected individuals presented with an early onset myopathy characterised by generalised skeletal muscle weakness and the presence of internal nuclei and myofibrillar aggregates on biopsy.	15
*RYR3* ^a^	Recessive missense variants were identified in a patient with childhood-onset nemaline myopathy.	16

^a^Only in isolated probands. Additional cases/families are required to support *PPA2* and *RYR3* as congenital myopathy disease genes.

In addition to the discovery of novel disease genes, our ability to perform massively parallel sequencing via whole exome or targeted gene panels has resulted in unexpectedly large expansions of the genotype–phenotype correlations for some myopathy genes ( Table 2).

**Table 2.** Novel genotype–phenotype associations, 2015–2018.

Gene	Finding	References
*ACTA1*	Skeletal muscle from three severely affected patients with the same p.Asn94Lys variant had cytoplasmic bodies but no nemaline bodies. Further mutations were associated with distal myopathy and progressive facioscapuloperoneal myopathy.	17– 19
*CACNA1S*	Dominant and recessive mutations, both resulting in reduced protein levels, were identified to cause severe congenital myopathies.	20
*FLNC*	Four unrelated patients with cardiomyopathy, arthrogryposis, and a limb-girdle pattern of skeletal muscle weakness at birth or during the first year of life harboured *de novo* missense variants; three of these patients had p.Ala1186Val.	21
*SCN4A*	A number of families presenting with severe congenital myopathy and *in utero* onset harboured recessive loss- of-function mutations. Rare variants that altered the function of the encoded voltage-gated Na ^+^ channel were more recently identified in cases of sudden infant death syndrome.	22, 23
*TNNT3*	A homozygous splice-site variant was identified in a single patient with profound skeletal muscle weakness, hypotonia, contractures, and nemaline bodies.	24
*TTN*	The largest cohort of patients (30 patients and 27 families) with congenital titinopathy associated with bi-allelic nonsense, truncating, or splice-site variants was recently described. All patients had prenatal or congenital onset hypotonia or contractures (or both), and almost half had cardiac involvement. One-third of the cohort harboured variants within meta-transcript-only exons, encoding a region of titin not found in the recognised mature skeletal muscle isoform transcript. ** These exons are thought to affect foetal titin transcripts and implicate developmental titin isoforms in disease pathogenesis.	25
*TRDN*	Recessive frameshift mutations, leading to loss of TRDN, were found to cause a skeletal myopathy in a subset of patients with triadin knockout syndrome.	26, 27

Therefore, the clearly defined entities are blurring into a continuum of myopathic phenotypes ^1^. This, coupled with the decreased use of skeletal muscle biopsies and corresponding identification of distinct pathological features, means that it is becoming increasingly common to refer to diseases by the causative gene (for example, actinopathies and titinopathies).

Mutations in key excitation–contraction coupling proteins have long been known to result in severe congenital myopathies (reviewed in 28), but the identification of mutations in *SCN4A* expanding the phenotype to sudden infant death syndrome (SIDS) ^22^ makes it tempting to speculate that functional variants in *RYR1* and *CACNA1S* are also responsible for a proportion of SIDS cases.

## Developments in molecular diagnostics

Definitive genetic diagnosis for the congenital myopathies is critical to the family for reproductive planning and for optimal care of the patient. Molecular diagnostics through massively parallel sequencing—whether by targeted gene panels or whole exome or genome sequencing—is becoming commonplace. While many extol the virtues of exome or genome sequencing, it is our opinion that currently targeted gene panels ^29, 30^ represent the “sweet spot” for molecular diagnostics for a number of reasons. It is our experience, and it is reported in the literature ^30^, that large gene panels are the most effective for genetic diagnosis because of varying degrees of clinical acumen; genetic overlap between different subtypes of neuromuscular disease; and the ever-expanding genotype–phenotype associations. These include improved read depth of the target region for the same or reduced sequencing cost ^31^, allowing the detection of variants in triplicated regions (this is particularly important for *NEB* and *TTN* ^25, 29, 32– 34– 16^); minimising incidental findings; reduced data handling and storage requirements; and better copy number variation calling compared with whole exome sequencing. Critical to accurate molecular diagnosis is the curation of variants by expert diagnosticians with an intimate knowledge of the group of diseases and their causative genes and proteins. Diagnostic centres where this is not the case sometimes miss pathogenic variants.

In support of the hypothesis that for many patients the causative mutation is an unrecognised mutation in a known disease gene, Cummings *et al*. obtained a 35% diagnostic rate by transcriptome-sequencing a cohort of patients that did not have a diagnosis following massively parallel sequencing of genomic DNA ^35^. We believe that inclusion of RNA sequencing (RNA-seq) in diagnostic workflows, for unsolved cases after whole exome or gene panel sequencing, is likely to result in improved diagnostic rates for congenital myopathies. A muscle biopsy can be readily collected for unsolved patients and therefore RNA-seq likely represents a better use of resources than moving to whole genome sequencing at this time.

A number of tertiary centres have investigated the place of rapid whole exome or genome sequencing within neonatal and paediatric intensive care units (ICUs). One such study recently abandoned the randomised standard testing arm because of loss of equipoise when it became apparent that rapid whole genome sequencing of trios resulted in more timely accurate genetic diagnosis of critically ill newborns ^36^. Given the high rate of *de novo* variants within the congenital myopathies, cases will continue to be encountered within ICUs despite the best preventative strategies (see section below on carrier screening for congenital myopathies). The high rates of genetic diagnosis in very sick babies in these ICU settings compared with cohorts with later-onset or milder diseases (or both) suggest that the underlying causes of disease in later-onset cohorts may not be purely Mendelian.

## Functional analysis

The use of animal models remains critical to the functional evaluation of novel variants and genes. However, with the increasing rate of variant discovery, functional analysis is becoming a bottleneck in both gene discovery and diagnosis. Selection of the most suitable models can reduce the time required. Examples include the recent analysis of variants in *PPA2* ^13^ and *PYROXD1* ^15^ using yeast and both yeast and zebrafish, respectively *.* Advances in CRISPR-Cas9 gene editing will allow the generation of sophisticated cell and animal models precisely mirroring changes observed in patients and will be of particular use in the analysis of large proteins (for example, *TTN*), where transgenic approaches are not feasible. While CRISPR-Cas9 gene editing is able to delete genes of interest relatively easily, generating specific point variations is much less efficient and takes considerable time and resources. In addition, it has been suggested that the best preclinical model for the evaluation of CRISPR-Cas9 gene therapies likely consists of patient cell lines, since the genomic background is identical to that of the patient. Thus, there will always be a place for patient biopsies and cell lines in research, and biobanking should continue to be supported for the congenital myopathies.

Whilst functional analysis of variants in novel disease genes is of great interest both diagnostically and for fundamental research, “variants of unknown significance” (VUSs) in known disease genes are a significant issue for diagnostic laboratories. However, the analysis of VUSs is less advantageous for research-focused laboratories. In our opinion, there is an urgent need for high-throughput functional genomics to be built into diagnostic pipelines and included in the cost of diagnosis. Diagnostic pipelines cannot continue to rely on research funding in research laboratories to perform functional analysis of VUSs in known disease genes.

## Pathophysiology of the congenital myopathies

There have been significant increases in the understanding of the pathophysiology of the major classes of the congenital myopathies, core myopathies, nemaline myopathies, and centronuclear myopathies. Recent large comprehensive reviews should be accessed for the current state of knowledge. These include reviews of the sarcomeric pathobiology that is the basis of the nemaline myopathies ^37^ and of the excitation–contraction coupling basis of the core and centronuclear myopathies ^28^. A 2015 review by Ravenscroft *et al*. ^38^ explored the overlap and blurring of the boundaries of the pathobiology of the different congenital myopathies, suggesting possible treatments. However, overall, our understanding of pathophysiology is not keeping pace with the discovery of new genes. Therefore, in this age in which journals demand more and more functional analysis in order to publish novel gene–disease relationships, it is prudent to remember that, after decades of research, sometimes the precise pathophysiological basis of a disease remains obscure. For example, the exact pathological mechanism of how superoxide dismutase 1 ( *SOD1*) mutations cause familial amyotrophic lateral sclerosis is not known a quarter of a century after the association was published ^39^. Similarly, the pathobiology by which mutations in the slow skeletal muscle/beta cardiac myosin ( *MYH7*) tail that breaks the coiled-coil rule can cause a distal myopathy is not known 14 years after the initial publication ^40^. However, even in the absence of a complete understanding of the pathophysiology, the identification of causative mutations has immediate benefits for diagnosis, counselling, and family planning. For genes in which the pathophysiological basis of the disease is identified, evidence-based approaches to therapy can be researched.

## Advances in therapies for congenital myopathies

A range of therapeutic options is under investigation and showing great promise for neuromuscular disease. These were recently reviewed by Dowling *et al*. ^41^. Genetic therapies for neuromuscular diseases are gaining much attention, most notably with the controversial US Food and Drug Administration (FDA) approval of exon skipping therapy for Duchenne muscular dystrophy ^42, 43^ and FDA approval of Spinraza (nusinersen) for spinal muscular atrophy. However, as the costs of these treatments are in the order of hundreds of thousands of dollars per patient per year, this renders them unaffordable for most families and leaves healthcare systems with difficult decisions to make ^45, 44, 29^. As such, the need for treatments that are more affordable persists.

A longer-term alternative to exon skipping would be genetic correction, but, to date, no studies have been published testing CRISPR-Cas9 gene editing approaches for the treatment of any congenital myopathy animal model. Success has been reported in models of muscular dystrophy ^46– 48^. However, in these studies, splice sites were disrupted, resulting in exon skipping and restored function. Though highly encouraging, this approach is not necessarily suited to the underlying molecular cause of most congenital myopathies, and the ability to efficiently correct point mutations *in vivo* is a significant hurdle.

Whereas genome editing is not likely to be clinically applied in congenital myopathy in the near future, other genetic approaches are perhaps closer, and there has been proof of principle in animal models. Lindqvist *et al*. delivered atrial/embryonic myosin light chain 1 (MyLC1 _a/emb_; encoded by *MYL4*) using AAV to the tibialis anterior of KI *Acta1* ^H40Y^ mice in an attempt to improve muscle force ^49^. *MYL4*-treated muscles were hypertrophic and had a marked increase in steady-state isometric maximal force production ^49^. It remains to be seen whether systemic delivery of *MYL4* in males of this line can rescue the early lethality caused by urethral obstruction ^50^.

Building on previous positive results with mouse and dog models of X-linked myotubular myopathy (XLMTM) ^51, 52^, the efficacy of systemic intravenous AAV delivery of canine myotubularin ( *Mtm1*) to dogs at 10 weeks of age (and already manifesting XLMTM) was assessed. Not only was the intervention well tolerated but also it corrected skeletal muscle pathology body-wide; improved neurological function, respiratory function, gait, and limb strength; and prolonged the usually shortened lifespan ^53^. Moreover, follow-up at four years post-treatment for two dogs demonstrated findings similar to those of unaffected littermates for multiple parameters ^54^. Most excitingly, a phase I/II clinical trial is under way for XLMTM using a single intravenous dose of AAV8 *hMTM1* (ASPIRO trial; ClinicalTrials.gov Identifier: NCT03199469). These advances for AAV approaches are also significant because of possible translation to other congenital myopathies.

Additional encouraging approaches for the centronuclear myopathies include (a) targeting dynamin 2 (usually upregulated in skeletal muscles from patients and mouse models with myotubular myopathy) with a short hairpin RNA ^55^ or an antisense oligonucleotide ^56^; (b) silencing mutant dynamin 2 in autosomal dominant centronuclear myopathy using allele-specific small interfering RNA (siRNA) sequences ^57^; (c) a spliceosome-mediated RNA trans-splicing strategy for dynamin 2 ^58^; (d) viral delivery of *Mtmr2*, a close homologue of the causative disease gene *MTM1* ^59, 60^; and (e) lowering phosphatidylinositol-3-phosphate accumulation through PIK3C2B inhibition ^61^. Centronuclear myopathy or other congenital myopathy patients with a confirmed *RYR1* mutation are being included in a current clinical trial to evaluate the clinical benefit of antioxidant therapy in RYR1 myopathy via thrice-daily oral/G tube n-acetylcysteine (ClinicalTrials.gov Identifier: NCT02362425). This follows promising preclinical data in *RYR1* cell lines and a zebrafish model ^62^.

Alongside the exploration of genetic approaches, more traditional and rapidly translatable approaches have been evaluated in animal models for nemaline myopathy. Although nemaline bodies are the hallmark pathological feature of nemaline myopathy, small myofibres are also common, suggesting that promoting muscle growth could be beneficial. Inhibiting myostatin in the KI *Acta1* ^H40Y^ nemaline myopathy mouse line using an activin type IIB receptor monoclonal antibody (ActRIIB-mFc; Acceleron Pharma, Cambridge, MA, USA) extended the usually shortened lifespan of male mice but did not improve other disease features ^50^. Myostatin inhibition treatment using mRK35 (Pfizer, New York, NY, USA) was investigated in the Tg *ACTA1* ^D286G^ mouse model of nemaline myopathy and was found to be efficacious in normalising body weight, myofibre force, and grip strength ^63^.

Patients with nemaline myopathy, their families, and clinicians have reported benefit for certain dietary supplements (for example, ^64, 65^ and a previous study with mice ^66^ were supportive). However, supplements did not improve muscle strength in animal models of nemaline myopathy: L-tyrosine in mouse and zebrafish skeletal muscle alpha-actin models ^67^ and creatine, L-tyrosine, L-carnitine, and taurine in a zebrafish nebulin model ^68^.

It has been observed previously that patients lacking skeletal muscle alpha-actin (ACTA1) retained high levels of the cardiac (foetal) actin isoform (ACTC1) in skeletal muscle and that the degree of ACTC1 expression determined the level of severity ^69^. Experimentally, overexpression of ACTC1 is able to rescue *Acta1* knockout mice ^70^. Recently, we described that the loss of the predominant alpha-actin in zebrafish resulted in a very mild phenotype because of compensatory upregulation of actin paralogues ^71^. Intriguingly, this compensation was triggered by mutation in the *actc1b* gene but did not result following knockdown of Actc1b, suggesting that the trigger was not the loss of actin protein but something intrinsic to the mutated gene or message. Determining the trigger for this compensatory response may allow a similar response to be induced in the case of *ACTA1* myopathy, resulting in compensatory ACTC1 expression and reduced disease severity.

## Carrier screening for congenital myopathies

Reproductive carrier screening programs for recessive diseases have been in place for nearly 50 years, starting with Tay–Sachs disease in the 1970s ^72^. Carrier screening aims to facilitate informed reproductive decision making by identifying those couples at risk of having an affected child with an (autosomal or X-linked) recessive disorder ^73^. As congenital myopathies are often severe diseases, they can be included in carrier screening programs (for example, nebulin [ *NEB*]-related nemaline myopathy) ^74^. Recommendations on carrier screening from professional associations have been evolving toward recommendations for broader screening. The American College of Obstetricians and Gynaecologists (ACOG) 2017 Opinion 690 on carrier screening recommends that a carrier screening approach be “offered to and discussed with each patient, ideally before pregnancy” ^75^. Israel has a nationwide panethnic carrier screening program in place, screening over 60,000 individuals a year for multiple conditions ^76^. In other countries, such as Australia, community interest in carrier screening is being investigated ^77^. The Australian Federal Government, in its 2018 budget, announced a $20 million research project into population-based reproductive carrier screening ( http://www.health.gov.au/internet/budget/publishing.nsf/Content/budget2018-factsheet65.htm). Although these programs will not remove the need for therapies, they could have profound impacts on the frequency, morbidity, and mortality of congenital myopathy.

## Conclusions

Congenital myopathy genetics, molecular diagnostics, pathophysiology, treatments, and prevention are advancing rapidly. The next few years, moving from gene discovery toward understanding pathophysiology and developing therapies, will see a need for changing skill sets and even broader multi-disciplinary teams to be involved in congenital myopathy research.
